# Autostereoscopic 3D Measurement Based on Adaptive Focus Volume Aggregation

**DOI:** 10.3390/s23239419

**Published:** 2023-11-26

**Authors:** Sanshan Gao, Chi Fai Cheung

**Affiliations:** State Key Laboratory of Ultra-Precision Machining Technology, Department of Industrial and Systems Engineering, The Hong Kong Polytechnic University, Hong Kong SAR, China; benny.cheung@polyu.edu.hk

**Keywords:** 3D measurement, autostereoscopic metrology, convolutional neural network, machine learning

## Abstract

Autostereoscopic three-dimensional measuring systems are a kind of portable and fast precision metrology instrument. The systems are based on integral imaging that makes use of a micro-lens array before an image sensor to observe measured parts from multiple perspectives. Since autostereoscopic measuring systems can obtain longitudinal and lateral information within single snapshots rapidly, the three-dimensional profiles of the measured parts can be reconstructed by shape from focus. In general, the reconstruction process consists of data acquisition, pre-processing, digital refocusing, focus measures, and depth estimation. The accuracy of depth estimation is determined by the focus volume generated by focus measure operators which could be sensitive to the noise during digital refocusing. Without prior knowledge and surface information, directly estimated depth maps usually contain severe noise and incorrect representation of continuous surfaces. To eliminate the effects of refocusing noise and take advantage of traditional focus measure methods with robustness, an adaptive focus volume aggregation method based on convolutional neural networks is presented to optimize the focus volume for more accurate depth estimation. Since a large amount of data and ground truth are costly to acquire for model convergence, backpropagation is performed for every sample under an unsupervised strategy. The training strategy makes use of a smoothness constraint and an identical distribution constraint that restricts the difference between the distribution of the network output and the distribution of ideal depth estimation. Experimental results show that the proposed adaptive aggregation method significantly reduces the noise during depth estimation and retains more accurate surface profiles. As a result, the autostereoscopic measuring system can directly recover surface profiles from raw data without any prior information.

## 1. Introduction

Precision metrology is of significance to the machining quality for precision manufacturing. Contact measuring systems make use of a stylus installed in a Coordinate Measuring Machine (CMM) to physically scan target surfaces to sample profile data. Non-contact systems mostly take advantage of optical instruments or sensors to acquire 3D information of measured parts. Due to their faster speed and contactless nature, non-contact systems play important roles in the precision measurement field. Popular non-contact measuring systems are based on interferometry [1], structured light [2], confocal microscopy [3], autostereoscopy [4], etc. Based on the 3D profiles acquired by these systems, data analysis studies including surface topography analysis [5], thickness characterization [6], defect detection, and segmentation [7] are performed to achieve more accurate measurement and characterization.

Due to the rapid speed of data acquisition, ease of implementation, and portability [4], autostereoscopic 3D measuring systems based on the integral imaging theory have emerged as a precision on-machine profile measurement method. To further improve measurement performance, disparity patterns [8] based on the parallax information of the recorded data were presented to enhance the accuracy of depth estimation. Methods based on epipolar space [9], point spread function [10], and structured light [11] were proposed for a more accurate depth reconstruction from the measurement data. Research on the elimination of specular light [12] and enhancement of data resolution [13] was performed to strengthen the quality of acquired data.

**Figure 1:**
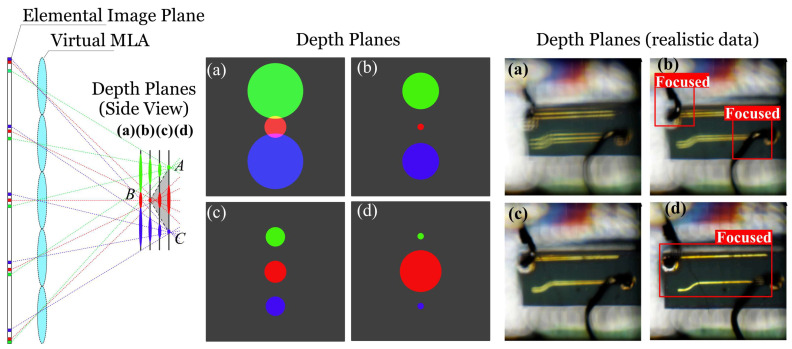
Digital refocusing using the measurement data recorded by the autostereoscopic measuring system. A and C are two points at the same depth. B is a point at a different depth. (a) Points A, B, and C are out of focus. (b) Point B is in focus. (c) Points A, B, and C are out of focus. (d) Points A and C are in focus.

The autostereoscopic measurement method makes use of a microlens array (MLA) in front of an image sensor to detect targets from multiple perspectives so that redundant parallax information of the targets can be recorded into elemental images within one snapshot. Due to the various observation perspectives of the microlenses, any one point of the target will be recorded multiple times in different elemental images behind the microlenses. The points reflecting the same target point are called corresponding points. As shown in Figure 1, the corresponding points in different elemental images are coloured in the same colour. The coordinate differences between the adjacent corresponding points are the disparity, which is definitely determined by the depths of the target points.

Shape from focus is an effective method to retrieve disparity from multi-perspective elemental images. In general, depth retrieval based on shape from focus is to detect the focus point in digital-refocused images. A focus measure operator is generally used to process the refocused images for the generation of a volume of focus measurements. The depth can be directly retrieved from the focus volume based on the winner-take-all (WTA) strategy. Various focus measure operators were developed to detect the focus points, including the Sobel gradient operator, Laplacian-based operators, the gray-level-variance operator, adaptive operators with flexible window sizes [14], and filters based on discrete cosine transform [15]. Since noise is inevitable during recording, refinement and volume aggregation are required to diminish the effects of noise in the focus volume. However, the robustness of the measure operators and aggregation methods is more challenging for the refocused images of the autostereoscopic measuring systems because of the limited resolution of the autostereoscopic data and the noise sensitivity of the systems.

Traditional focus measure operators such as the Sobel operator and Laplacian-based operator are robust for most scenes, albeit high-frequency noise will affect the accuracy of focus detection. However, it is difficult to train a learning-based focus measure method with satisfying the generalization capabilities for autostereoscopic measurement since the measurement data are limited, and slight changes to the recording conditions could result in large differences in autostereoscopic imaging. Hence, it is important to seek an adaptive manner to achieve more accurate estimation and measurement on the basis of the integration of existing and learning methods to maximize the advantage of traditional methods. In this paper, a method for adaptive focus volume aggregation is presented to diminish the noise produced during digital refocusing so as to realise more accurate depth estimation. The proposed method exploits a convolutional neural network (CNN) model to adaptively aggregate the focus volume generated from focus measures. The model is constrained with a smoothness constraint and an identical distribution constraint during backpropagation. The identical distribution constraint restricts that the refined results of the proposed network and the preliminary results generated by traditional methods are sampled from the same ideal distribution of the desired depth map. As a result, the network is able to approach the ideal distribution to generate more accurate results. The weight optimization of the proposed model is performed in an unsupervised fashion for every measured sample so that no establishment of datasets is required. Experimental analysis reveals that the adaptive aggregation method is effective in reducing the effects of the refocusing noise. More accurate depth estimation with smooth surfaces and sharp edges is obtained after the proposed aggregation.

## 2. Shape from Focus

Shape from focus is a method that retrieves the geometrical information of the target samples from refocused image stacks. The raw measurement data, which are also called elemental images, store corresponding points regarding a target point. Hence, the disparity can be acquired via the determination of the coordinate differences between the neighbouring corresponding points. Digital refocusing simulates the inverse process of recording, virtually placing an MLA behind the elemental image plane so that the corresponding points focus again on different depth planes. Based on the refocusing concept, refocused images are easily acquired, and the corresponding points are solely focused in the refocused image at the correct depth. Figure 1 illustrates the blue, red, and green points in different elemental images corresponding to target points A, B, and C. The distances from the recording lens to these target points differ. Passing through the virtual MLA, these corresponding points focused on different depth planes. The corresponding points to B focus on (b) and those to A and C focus on (d). As a result, a refocused image stack is acquired through projecting these corresponding points to different depth planes. An example of a refocused image stack is exhibited on the right of Figure 1, where the points closer to the lens focus on (b) and those farther to the lens focus on (d).

Focus volume is obtained through applying a focus measure operator to the refocused image stack. After focus measures, a box filter is usually used to aggregate the acquired focus volume. This is equivalent to local aggregation on focus volumes [16]. A coarse depth map can be obtained from the aggregated focus volume through the winner-take-all (WTA) strategy. One all-in-focus image that contains all focused visible contents can be recovered using the coarse depth map and the refocused image stack. Eventually, a guided filter can be used to filter the coarse depth map and the all-in-focus image to obtain a refined depth map with smooth patterns.

### 2.1. Adaptive Focus Volume Aggregation

**Figure 2:**
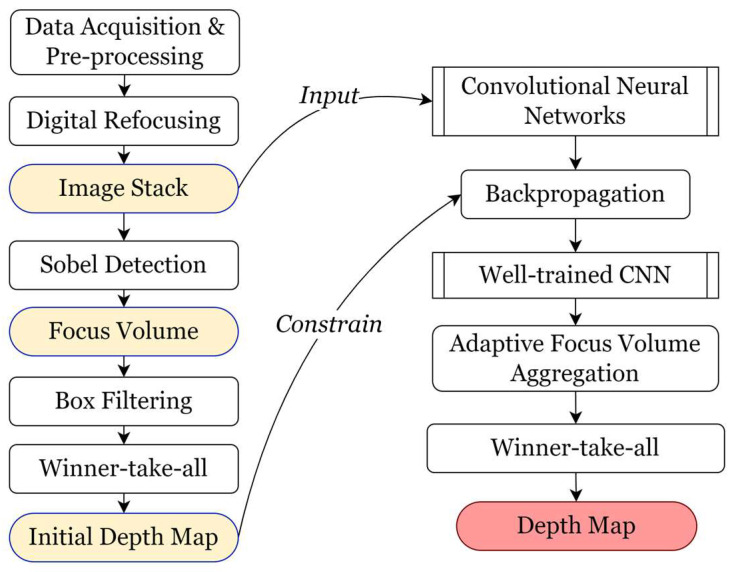
Adaptive focus volume aggregation using a convolutional neural network.

On the basis of shape from focus, the proposed method for adaptive aggregation first recovers an initial depth map and an initial all-in-focus image following the procedure as shown in Figure 2. Data recorded by the autostereoscopic sensor are going through calibration, trimming, preliminary denoising, and pre-enhancement. Digital refocusing is conducted on the pre-processed autostereoscopic data to generate a stack of refocused images at different depth planes. The Sobel measure operator is used for focused points detection, which separates the high-frequency signals with apparently high gradients for focus detection. Although other filters such as Laplacian-based operators can also access similar results, the noise produced during refocusing from autostereoscopic data usually introduces the incorrect detection of focused parts. For instance, a focused point detected by measure operators could actually be high-frequency noise. A focus volume is obtained after the focus measure process. The box filter is used for a preliminary aggregation to the focus volume so that initial depth estimation can be acquired through the WTA strategy. The initial estimation will be utilized in the unsupervised backpropagation process. After applying guided filtering, a refined depth map based on the Sobel measure operator is acquired.

In terms of the adaptive aggregation process, a convolutional neural network is built to achieve focus measurement and volume aggregation. The input of the network is the refocused image stack recovered from digital refocusing. The focus measure of the network is guided by the Sobel operator. The adaptive aggregation is automatically realised via a series of 3D convolutional kernels. During training, the network adaptively optimizes its weights via backpropagation under the constraint of the initial depth map. After iterative learning, a well-trained CNN model is obtained and is capable of realizing accurate focus volume aggregation adaptively for the measured sample. Similarly, depth estimation and its corresponding all-in-focus image are acquired via the WTA strategy after obtaining the adaptively aggregated focus volume.

### 2.2. Aggregation Network and Unsupervised Learning

**Figure 3:**
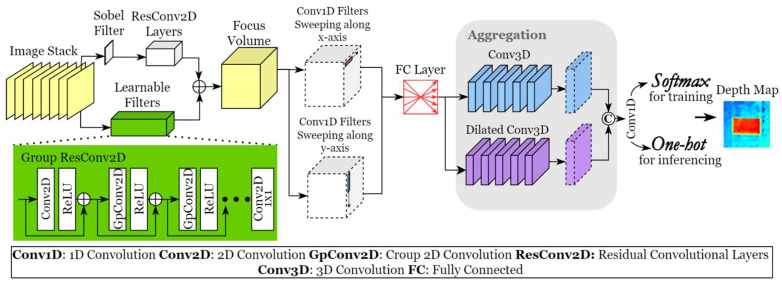
Framework of the proposed aggregation network.

The network framework is shown in Figure 3. To accelerate the learning speed, the Sobel operator is used to produce guidance focus measurement from the refocused image stack. A series of learnable filters are constructed using residual convolutional layers to detect the focused contents of the input. Group convolution is used for the focus measurement so that each feature map can be solely convolved by the filters in one group. As a result, the filtering results of every convolutional group will not affect each other during the forward process. The last layer of the learnable filters is 1 × 1 2D convolutional kernels to integrate the filtering results of different convolution groups into one focus measure. Another convolutional block composed of a series of 2D residual convolutional layers is used to further enhance the detection results of the Sobel operator. In addition, the block makes the output of the learnable filters and the Sobel filter have the same scalar. Then, the focus measure acquired by the learnable filters is further pixel-wisely integrated with the convolution results of the Sobel-based focus measure. As a result, a focus volume guided by the Sobel operator is obtained.

The focus measure operator always highlights high-frequency signals such as edges and distinct patterns to detect focused parts. This may cause the focus measure to be discontinuous. In the proposed method, multiple convolutional kernels with one dimension are used to sweep the focus volume along the x-axis and the y-axis. The line-by-line sweeping contributes to the refinement of the discontinuous detection so that smoother estimation can be realised. During the sweeping, instead of considering points in a 2D grid, only neighbouring points within a line are taken into consideration to refine discontinuity. The large estimation bias of a point will be eliminated through weighting the neighbouring points’ value. A large 1D kernel is prone to remove salient features to guarantee excessive smoothness, which was also observed during experiments. Hence, only two-layer 1D kernels with limited receptive fields are used for the sweeping. The output of the two-axis sweeping is fused by fully connected layers to obtain a refined focus volume. A series of 3D convolutional kernels are used for subsequent volume aggregation since the 3D kernels take the information within a 3D square domain of the focus volume into account. This enables aggregation to take place not just between neighbouring points of a single focus measure but also among neighbouring measures corresponding to different depth planes. It is noted that different window sizes, i.e., the receptive fields of convolutional kernels, focus on different information. A small kernel pays more attention to local information for retaining more details of geometries, whereas a kernel with a large receptive field usually focuses on global information and high-level features, contributing to the representation of the continuity of the targets. To take advantage of the different receptive fields, two aggregation sub-networks consisting of 3D convolutional layers are used parallelly to process the refined focus volume. Taking the learning complexity into consideration, dilated convolutional kernels are used to achieve large receptive fields, instead of applying large kernels with more weights or deeper layers.

The output of the two aggregation sub-networks is concatenated and integrated by 1 × 1 convolutional kernels. As a result, an aggregated focus volume is obtained for depth estimation. In addition, the winner-take-all strategy is replaced by Softmax as an approximation so that the whole process is derivable during the backpropagation process. To improve the learning stability of Softmax, the gumbel-Softmax method is used during the training process and the temperature coefficient is set to 1. One-hot mapping, which is not derivable, is used during the inference process to replace Softmax so as to output more accurate results.

**Figure 4:**
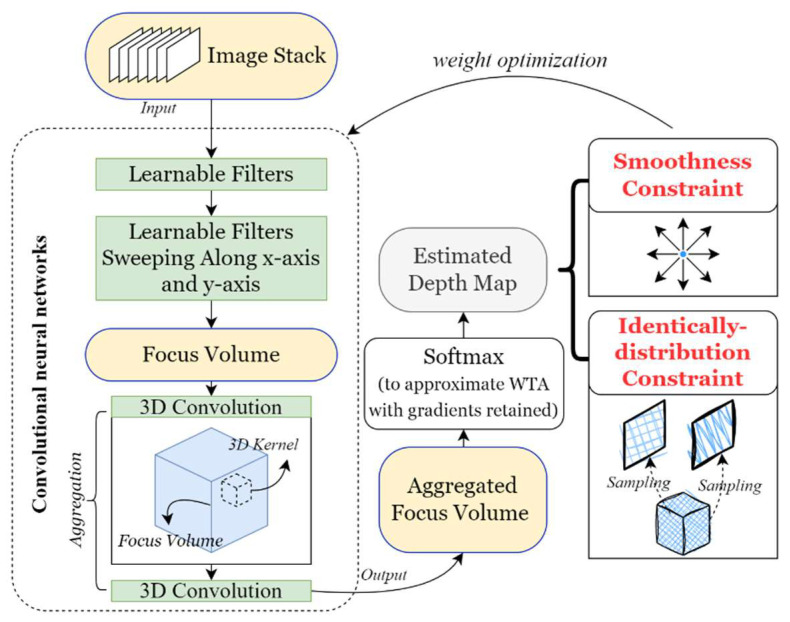
The proposed method and the learning process.

A problem in training the proposed network is the lack of ground truth for depth estimation. Unsupervised learning, therefore, is used for the weight optimization of the network, as shown in Figure 4. Assuming that the desired high-accuracy depth map X is sampled from one ideal Gaussian distribution P(X)=𝒩(μ,Σ) that can describe the depth information of the current measured part, the depth map $X^{r}$ generated using the adaptive aggregation network can be considered a sampling result of $P\left(X^{r}\right)$. The coarse initial depth map $X^{0}$ estimated directly from the preliminary focus volume can be also sampled from the ideal distribution, but with noise. It is assumed that the noise also follows a Gaussian distribution ε∼𝒩(0,I). The initial depth map can be re-parameterized as

(1) $$X^{0}=\mu \left(X\right)+\Sigma^{\frac{1}{2}}\left(X\right)\times \varepsilon$$

where μ(X) and Σ(X) are the mean and covariance of P(X), respectively. The target of the learning is to produce a distribution infinitely approaching the ideal distribution P(X), i.e., $P\left(X^{r}\right)\approx P\left(X\right)$. Hence, the identical distribution loss can be defined as

(2) $$L_{ID}\left(\alpha \right)=\sum_{i}\left(\left\|\varepsilon_{i}\times \alpha +X_{i}^{r}-X^{0}\right\|\right)$$

where α is the scalar of the noise. During training, in every epoch, the coarse preliminary depth map $X^{0}$ is resampled via interpolation to simulate coarse distribution.

Another smoothness constraint is used to suppress the gradients of the final depth map so that the regions representing surfaces are estimated more smoothly, which is expressed as

(3) $$L_{smooth}=\sum_{i}\left(\nabla_{x}X_{i}^{r}+\nabla_{y}X_{i}^{r}\right)$$

The total training loss is Equation (4), where β is the penalty factor of the smoothness constraint. Under the constraints, the proposed model can be trained via backpropagation in an unsupervised fashion for each sample separately, without the need for dataset construction.

(4) $$L=L_{ID}\left(\alpha \right)+\beta L_{smooth}$$

## 3. Results and Discussion

**Figure 5:**
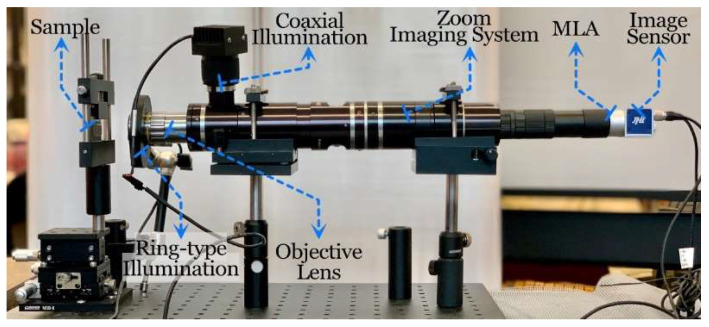
Setup of the autostereoscopic measuring system.

The setup of the autostereoscopic measuring system is shown in Figure 5, where an objective lens mounted on a zoom imaging system records the measured sample, and an MLA is inserted in front of an image sensor to observe the target from different perspectives. Ring-type illumination and coaxial illumination are used in the system to light the measured microstructures. The specifications of the system are exhibited in Table 1.

Preliminary experiments found that the learnable filters with deeper layers that contribute to large receptive fields are more effective for focus measurements, whereas deeper layers can increase learning complexity. Hence, the learnable filters are constructed with five residual group convolutional layers, with 3 × 3-size kernels and eight groups. Moreover, the large receptive fields of the kernels for sweeping may sacrifice more details for smoothness. As a result, two layers of 1D convolutional kernels with size 3 are used for refinement. Two aggregation sub-networks both contain seven 3D convolutional layers, where the first layer is used to increase dimension and the last layer works for suppressing the dimension. The dilation size for the large receptive field aggregation sub-network is set to 3. The Rectified Linear Unit (ReLU) is used as the activation function of the proposed network. During training, α and β are set to 0.25 and 1.0, which are determined through grid search. For faster learning, the channel number is set to 8 and only 100 epochs are used for training. The running time for one inference process of the network is 0.827 s on average using an Nvidia GeForce RTX 2080. The model is initialized under a Kaiming normal distribution [17]. 

**Figure 6:**
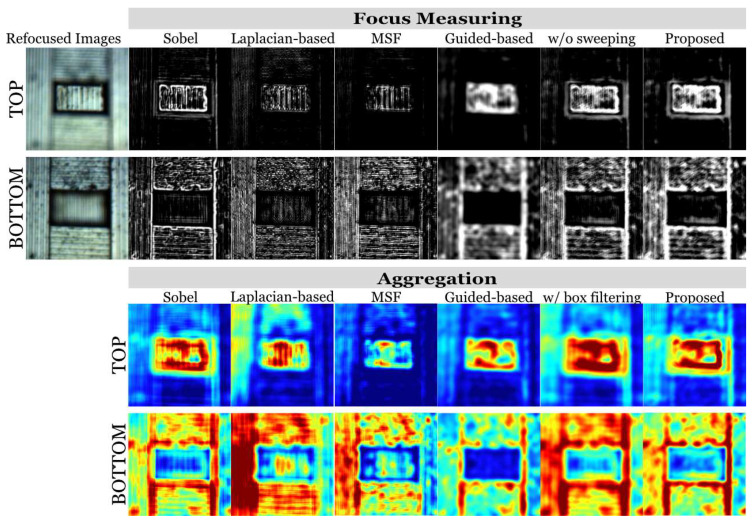
Visualization of focus measurement and aggregation by the Sobel operator, the Laplacian-based operator, the guided-based filter, the proposed filter with box filtering, and the proposed filter with adaptive aggregation.

**Figure 7:**
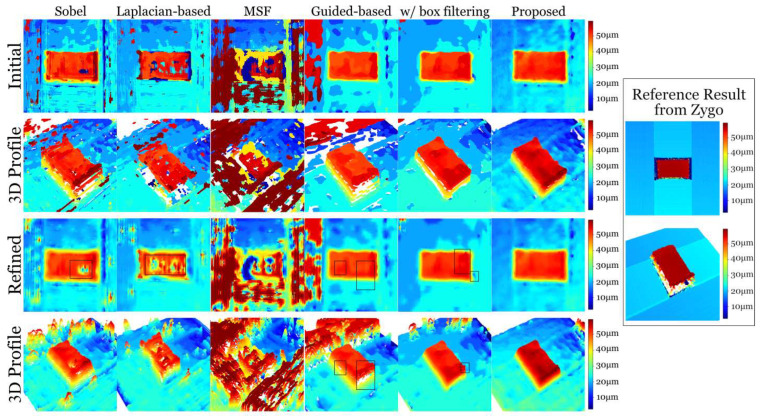
Results of depth reconstruction based on various methods. The results are produced by various methods, including Sobel, a Laplacian-based operator, a multi-scale focus (MSF) measure, a guided-based measure, the proposed method with box filtering, and the proposed method using adaptive aggregation. The initial estimation and refined results are displayed, accompanied by their corresponding 3D profiles of the measured surface.

Analyses of focus measure and aggregation results based on different focus measure operators are shown in Figure 6. Generally used operators, including the Sobel operator, the Laplacian-based operator, a multi-scale focus (MSF) measure [18], and a guided-based measure [19], were applied in the experiments for comparison and the proposed aggregation results with a box filter are demonstrated for qualitative analysis. It was found that traditional focus measures, such as the Sobel filter and the Laplacian-based filter, can detect the focused key points in the images and retain distinct high-frequency information. However, noise effects resulting from image artefacts during refocusing are severe in the focus measure results and the aggregation results. In addition, discontinuous detection also results in sparse aggregation results on the continuous surface, which generates unexpected holes or protrusions on the surface in the final estimation (see Figure 7). The guided-based filter generates more smooth detection results to guarantee the continuity of the estimation whereas the edge patterns, i.e., the high-frequency information is sacrificed. Since the MSF method is developed for macro-object images captured with traditional cameras, the measure fails to correctly score the focus parts of the autostereoscopic data. The focus measuring result performed by the 1D kernels sweeping along the x- and y-axis is shown in Figure 6, where the continuity of the detection is improved after sweeping so that the proposed method can achieve continuous and smooth estimation. Additionally, the focus measurement generated through the proposed method contains clear high-frequency patterns as the Sobel operator does. Regarding the aggregation processes exhibited in the lower part of Figure 6, conventional aggregation is more sensitive to the focus measure results. For instance, unexpected discontinuity and holes appear on the top after aggregation, and protrusions are generated on the bottom. This also can be found in the final 3D estimation results (shown in Figure 7). The edges and salient features cannot be aggregated correctly via the conventional process, which can be observed in the results produced via conventional aggregation and the proposed method. The two processes are both based on the learning-based focus measuring filters. However, the edges aggregated conventionally cannot retain the sharp features of the focus measurement results. This could result from averagely weighting all the neighbouring points, some of which have a negative contribution to correct profile reconstruction. The proposed adaptive aggregation method can retain clear edges and patterns.

The depth estimation results are shown in Figure 7. The initial results are directly estimated from the aggregated focus volume based on various methods, and the refined results are acquired through applying guided filters to the initial results and the corresponding all-in-focus images. A reference result obtained using a commercial measurement product—Zygo Nexview Optical Profiler—is exhibited in the figure. However, there are some missing points that cannot be measured using the product. The results based on Sobel and Laplacian contain severe holes and protrusions caused by discontinuous estimation, though they can be improved when the aggregation filter size increases. However, a large-size filter during aggregation often causes inaccurate edge and geometry estimation, since the large aggregation filter diminishes the contribution of the high-frequency signals, some of which are important to the reconstruction of geometries. The results based on the guided filter and the proposed filter with box filtering can retain smooth estimation. However, the edge information is lossy compared with the results from the Sobel operator. It is obvious that the result estimated using the proposed filters and the adaptive aggregation method is more accurate with reasonable smoothness. The reconstructed geometrical structure of the measured part is more accurate as well. It can be also found that the depth estimation of the measured surface tends to have smooth gradients compared with other results. 

**Figure 8:**
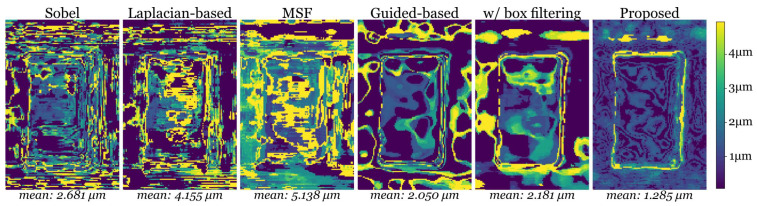
Standard deviation of repeated measurements based on various methods.

Repeated measurements were performed to evaluate the uncertainty of the proposed methods. For each measurement, the learning model was retrained without initializing pre-trained weights to evaluate the reproductivity of the model. The standard deviation of 10 repeated measurement results was acquired through the iterative closest point algorithm and is shown in Figure 8, where different methods based on the Sobel operator, the Laplacian operator, and the guided filter are compared. It was found that the Sobel filter and the Laplacian-based filter are sensitive to noise in different measurement scenes, so the standard deviation is larger when measuring the same objects. The guided-based method and the proposed filter aggregated using the box filter are able to produce smooth surfaces to diminish noise. However, the estimation of the surface has a large deviation in the results of the two methods. Although the proposed method uses a learning model, the results have considerably small deviations. This reveals the capability of the proposed method for repeatable precision measurements. Since the network is trained based on the coarse depth estimation result, coarse estimation with errors could result in large bias points in the final results. Hence, a dataset composed of more measurement data can eliminate the bias during training. On the basis of the dataset, the proposed network can be pre-trained to learn to represent more general features. The pre-trained network could be more robust after transfer learning on different samples.

## 4. Conclusions

Autostereoscopic 3D measuring methods acquire 3D information through retrieving depth from measurement data. Disparity information that is determined by the depth of the target parts can be recovered through shape from focus. The proposed adaptive focus volume aggregation method is capable of performing focus measurement and adaptively aggregating the focus volume generated from digital refocusing, on the basis of the guidance of traditional focus measure methods. A convolutional neural network and an unsupervised learning method for every measured part were presented to adaptively retrieve depth information from the refocused image stacks. A smoothness constraint and an identical distribution constraint were presented for the backpropagation process of the neural network to make the network learn to approach the ideal distribution of the desired depth estimation. Experiments showed that more accurate depth and profiles can be recovered after the adaptive aggregation compared with the direct depth retrieving from the refocused image stack. The proposed method integrates traditional methods with learning models effectively and shows its capability of effective volume aggregation for autostereoscopic measurement data. It is also possible to train a pre-trained model for adaptive focus measure and aggregation using a large amount of data to establish a more robust learning network for measured samples with different features.

## Figures and Tables

**Table 1:** Specifications of the autostereoscopic measuring system.

Item	Specification	
CCD Sensor	Pixel Size	3.45 μm
	Sensor Size	2/3 inch
	Resolution	2456 × 2058
MLA	Pitch	150 μm
	Focal Length	5.6 mm
	Scale	10 mm × 10 mm
Objective Lens System	NA	0.28
	Magnification	0.64–4.5

## Data Availability

The data presented in this study are available on request.
